# Key Factors Influencing the Mechanical Properties of Binodal Decomposed Metallic Glass Composites

**DOI:** 10.3390/ma18245593

**Published:** 2025-12-12

**Authors:** Yongwei Wang, Guangping Zheng, Mo Li

**Affiliations:** 1 Collaborative Innovation Center of Steel Technology, University of Science and Technology Beijing, Beijing 100083, China; 2 Department of Mechanical Engineering, Hong Kong Polytechnic University, Hung Hom, Kowloon, Hong Kong 10000, China; mmzheng@polyu.edu.hk; 3 School of Materials Science and Engineering, Georgia Institute of Technology, Atlanta, GA 30332, USA

**Keywords:** Binodal decomposed metallic glass composites, free volume theory, shear banding, size effect, strength

## Abstract

Structural heterogeneity plays a crucial role in enhancing the mechanical properties of metallic glasses (MGs) by impeding the propagation of shear bands (SBs). Metallic glass matrix composites (MGCs) consisting of reinforcements are of great interest as they enhance the mechanical performance of brittle MGs. However, managing the dispersity of hetero-phases within the glassy matrix presents technical challenges due to surface tension and thermal property incompatibility. Binodal phase separation is an effective approach for fabricating MGCs with uniformly dispersed glassy droplets or particles. The species of matrix and characteristics of particle reinforcements significantly influence mechanical properties. This study theoretically examines how the fraction, size, and variety of particle reinforcements influence performance using finite element models based on free volume theory. The synergistic mechanisms for performance tuning involve stress fields generated by particle reinforcements that modify the nucleation and propagation of SBs in the matrix. Additionally, the size effect of particles depends on their interaction with SBs. This comprehensive understanding could substantially enhance the design and optimization for MGCs.

## 1. Introduction

Metallic glasses (MGs) exhibit exceptional mechanical, magnetic, and catalytic properties due to their disordered atomic structure [1]. However, a significant drawback is their limited macroscopic plastic deformability at room temperature, caused by the formation of highly localized shear bands (SBs) [2]. Recent efforts have focused on enhancing the plasticity of MGs. The concept of heterogeneity, including composites, is widely recognized as beneficial for improving MGs plasticity by obstructing SBs [3,4,5,6,7,8,9,10]. Recently, metallic glass matrix composites (MGCs), which consist of reinforcements in a glassy matrix, are garnering considerable interest because they enhance the mechanical properties of brittle MGs [11,12,13,14,15,16,17,18,19]. The desired expect is that the second phase (such as metal, ceramic, metallic glass, etc.) should increase SBs nucleation while hindering their propagation [20,21,22,23,24,25,26,27,28,29]. This can significantly improve plasticity by controlling the homogeneous distribution of SBs [30,31,32,33,34,35,36,37,38]. For example, Hofmann [13] invented three in situ MGCs with dendritic crystal phase (Zr_36.6_Ti_31.4_Nb_7_Cu_5.9_Be_19.1_, Zr_38.3_Ti_32.9_Nb_7.3_Cu_6.2_Be_15.3_ and Zr_39.6_Ti_33.9_Nb_7.6_Cu_6.4_Be_12.5_), which exhibited volume fractions of 42%, 51%, and 67% for the dendritic phase, respectively. Their total strain to failure ranges from 9.6 to 13.1% at ambient temperature. The tensile ductility is associated with patterns of locally parallel primary SBs that form in domains defined by individual dendrites. The appropriate characteristic length scale of the crack tip’s plastic zone can limit shear band extension, suppress SB opening, and prevent crack growth. Liu et al. [28] synthesized the ZrCuNiAl MGs, which exhibit super compressive plasticity of 160% at room temperature, attributed to a microstructure of hard glassy regions surrounded by soft glassy regions. It was found that the SB nuclei preferentially form in soft regions, while their propagation is hindered by hard regions. This impedance changes the propagation directions and promotes SB multiplication. It is evident that the homogeneous distribution of secondary reinforcements plays a critical role in influencing SBs formation. However, managing the dispersity of hetero-phases within the glassy matrix presents technical challenges due to issues related to surface tension and incompatibility in thermal properties.

Recently, phase separation has emerged as a novel approach for producing in situ MGCs with the homogeneous dispersity of the second phase, whose size could range from nanometers to several tens of micrometers [29,30,31,32,33,34,35,36,37,38]. An interconnected-type structure (Spinodal decomposition) or a droplet-type structure (Binodal decomposition) can be obtained. There are strong atomic-level bonding interfaces between the secondary reinforcements and the glassy matrix. Experimental observations indicate that alloys with a high positive enthalpy of mixing and strong glass-forming ability can be separated into two distinct glassy phases during solidification. Under specific cooling conditions, the decomposed phases with varying chemical compositions can be frozen into the glassy phases which have varying mechanical properties and free volume (FV) density [18,19,20]. While the key factors critically influencing the mechanical responses of Spinodal decomposed MGCs have been thoroughly investigated [36], the critical factors affecting the performances of the Binodal decomposed metallic glass composites (BDMGCs) remain unidentified. In this works, using FV as a characteristic parameter to gauge variations in the microstructure and changes in chemical composition within the glassy phases, we systematically explore how heterogeneous structures modulate SB dynamics and mechanical performance of BDMGCs through finite element models (FEMs) that integrate the free volume theory.

## 2. Materials and Methods

**Figure 1:**
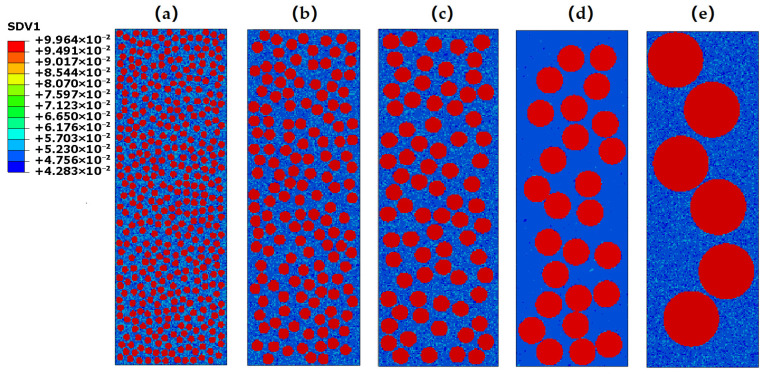
The initial FV spatial distribution of BDMGCs with a specific volume fraction *F* = 39% of particles, having $\rho_{p}$ = 0.10, and diameter *D* = 6 (**a**), 10 (**b**), 14 (**c**), 24 (**d**), and 50 (**e**) in matrix A.

As aforementioned, the BDMGCs characterized by a droplet-type microstructure, exhibit a uniform and homogeneous distribution of the spherical glassy reinforcements within the MG matrix. The variety, fraction, and size of isolated glassy particles, along with the type of MG matrix, significantly affect the mechanical properties of BDMGCs. To obtain the mechanical properties of BDMGCs with various mechanical properties of particles at the micro-scale, we utilize continuum mechanics to model the mechanical behaviors of the BDMGCs that contain the submicron-sized particles. The FEMs explicitly incorporate the FV variations as the microstructure parameter of MGs [39,40,41,42,43,44,45,46]. Each mesh element is assigned the FV values according to certain spatial morphology, such as those following the droplet-like structure (Figure 1). In FEMs, all elements are square in shape, with the side length specified as d = 0.1 μm. By varying the distribution of FV, i.e., $\rho_{M}$ and $\rho_{p}$, within the MG matrix and glassy droplets, respectively, we can create heterogeneous microstructures that exhibit the patterns of BDMGCs. The matrix type is defined by the random homogeneous spatial distribution of a specific statistical distribution of FV. The FV values in a matrix can be derived from statistical distributions transformed from a beta distribution, 0.04 × *Beta*(50,50) + 0.03, 0.04 × *Beta*(1,1) + 0.03, or 0.04 × *Beta*(0.1,0.1) + 0.03, which is labeled as matrix A, B, or C, respectively. The histogram of FV in matrix A resembles a truncated Gaussian, while matrix B displays a uniform distribution, and matrix C shows a bimodal distribution. The FVs in those matrices range from 0.03 to 0.07, with a mean value of 0.05 [43]. The BDMGCs samples, comprising matrix A, B or C, are labeled as systems MA, MB, or MC, respectively. The value of $\rho_{p}$ at the particles ranges from 0.02 to 0.10, allowing us to explore how the varying mechanical properties of reinforcements influence the mechanical properties of BDMGCs as the FV transitions from hard particle ($\rho_{p}$ = 0.02) to soft particles ($\rho_{p}$ = 0.10). Particle diameter *D* is measured by the number of elements across the particle, expressed as a multiple of *d*. To investigate the size effect of particles, the diameters *D* were chosen as 6, 10, 14, 24, and 50. The volume fraction *F* of particles was approximately set at three levels: 13%, 26%, and 39%. Since the total number of each model was fixed, for a given volume fraction *F*, the equivalent diameter *D* was inversely related to the number of particles. Consequently, as the number of particles increases, *D* decreases, and vice versa. The systems can be denoted for simplification: for example, “MA-*D*10$\rho_{p}0.02$*F*13” indicates that the diameter of the glassy particle with $\rho_{p}=0.02$ is 10 times mesh unit and the total volume fraction *F* is 13% in glassy matrix A.

As shown in previous works [43,44], these parameters enable a systematic and quantitative investigation of the effect of heterogeneous microstructure on mechanical properties of BDMGCs. To obtain the mechanical properties of all samples, we employed an elastoplastic, constitutive relationship that incorporates FV as a structural variable. The deformation strain in the models includes the elastic and plastic components:

(1) $$\varepsilon_{ij}=\varepsilon_{ij}^{el}+\varepsilon_{ij}^{pl}$$

where $\varepsilon_{ij}^{el}$ is elastic strain of an isotropic MG. The plastic strain can be derived from the plastic flow equation:

(2) $$d\varepsilon_{ij}^{pl}=d\lambda \frac{\partial g}{\partial \sigma_{ij}}$$

where *g* is the plastic potential function based on the Drucker–Prager yield surface,

(3) $$g(\sigma_{ij})=b^{\prime }I_{1}+\sqrt{3J_{2}}-K$$

where λ is the plastic deformation parameter related to FV change, $\sigma_{ij}$ is the Cauchy stress, and $I_{1}$ is the first invariant of the stress tensor $\sigma_{ij}$, and $J_{2}$ is the second invariant of the deviatoric stress. The effective shear stress and increment of equivalent plastic strain are expressed in the following relations:

(4) $$\sigma_{DP}=a^{\prime }I_{1}+\sqrt{3J_{2}}$$

(5) $$d\varepsilon_{eff}^{pl}=\sqrt{(2/3)d\varepsilon_{ij}^{pl}d\varepsilon_{ij}^{pl}}$$

where $a^{\prime }$, $b^{\prime }$ and K are constant and $a^{\prime }=b^{\prime }$ for the associated flow rule in our models. The plastic strain rate depends on the FV production and the effective shear stress. The plastic strain rate and the FV change rate are presented in the following relations based on the free volume theory, respectively:

(6) ε˙effpl=2fexp−αv∗v¯fexp−ΔGmkBTsinhσDPΩ2kBT

(7) ∂v¯f∂t=v∗fexp−αv∗v¯fexp−ΔGmkBT6αkBT(1−μ)v¯fEcoshσDPΩ2αkBT−1−1nD+κ∇2v¯f

where ${\bar{v}}_{f}$ is the mean FV, α is a geometrical factor close to 1, $k_{B}$ is the Boltzmann constant, T is the current temperature, $v^{*}$ is the hard sphere volume of the atom, Ω is the atomic volume, $\Delta G^{m}$ is the activation energy of atomic jump, f is the frequency of atomic vibration, $n_{D}$ is the number of atomic jumps needed to annihilate a FV equal to $v^{*}$, ranging from 3 to 10, *E* denotes the Young’s modulus and μ is Poisson ratio, and κ refers to a free volume gradient coefficient. Here we use the material properties of Zr_41.25_Ti_13.75_Ni_10_Cu_12.5_Be_22.5_ MG [42].

Equations (1)–(7) prescribe the constitutive relationship for the MG, without explicitly distinguishing between homogeneous and inhomogeneous deformation behavior. Using the finite element method, we can calculate displacements, strains, and stresses that satisfy the governing equations for a given load:

(8) $$\frac{\partial \sigma_{\mathrm{ji}}}{\partial x_{i}}+\frac{\partial \rho \nu_{i}}{\partial t}+\rho f_{i}=0$$

(9) $$\varepsilon_{\mathrm{ij}}=\frac{1}{2}(u_{i,j}+u_{j,i})$$

with the boundary condition is: ${\sigma^{*}}_{ij}n_{j}=t_{i}$, here the body force $f_{i}$ is zero, and the density change rate is ignored, i.e., $\frac{\partial \rho }{\partial t}$ = 0. Based on the variation principle, we transform the partial differential equations governing microscopic stress equilibrium (8) and the associated boundary conditions into a weak form of the integral equation:

(10) $$\int_{V}\sigma_{\mathrm{ij}}\delta \varepsilon_{ij}dV+\int_{\partial V}{\sigma^{*}}_{ij}n_{j}\delta v_{i}dS=0$$

in the FEM, the displacement value at any location within the solid mesh can be calculated by interpolating between values at mesh nodes through the shape function *N*. By substituting the interpolated shape function into the weak form of the integral equation, we obtain:

(11) $$\int_{V}\sigma_{ij}(\Delta \varepsilon_{\mathrm{kl}}(u_{i}^{a}))\frac{\partial N^{a}}{\partial x_{j}}dV+\int_{V}\frac{\partial \Delta \sigma_{ij}}{\partial \Delta \varepsilon_{\mathrm{kl}}}\frac{\partial N^{b}}{\partial x_{i}}\frac{\partial N^{a}}{\partial x_{j}}\Delta u_{i}^{a}dV+\int_{\partial V}{\sigma^{*}}_{ij}n_{j}N^{a}dS=0$$

we can rewrite the weak form of the integral equation as a system of linear equations. The material stiffness matrix for the constitutive models,

(12) $$D_{ijkl}^{ep}=\partial \Delta \sigma_{ij}/\partial \Delta \varepsilon_{kl}$$

is implemented in finite element software ABAQUS 6.13 through a UMAT subroutine [42,43,44]. In the implicit integration scheme, applying the backward Euler method to the flow Equation (6) and FV evolution Equation (7), we get:

(13) $$\sigma_{DP,(n+1)}^{trial}-\sigma_{DP,(n)}-\Delta \sigma_{DP}-(Kab+3G)\frac{1}{\sqrt{\frac{2}{9}b^{2}+1}}2\Delta t\exp(-\frac{1}{{\bar{v}}_{f(n)}+\Delta {\bar{v}}_{f}})\exp\left(-\frac{\Delta G^{m}}{k_{B}T}\right)\sin h(\frac{\sigma_{DP(n)}+\Delta \sigma_{DP}}{\sigma_{0}})=0$$

(14) Δv¯f−Δtαexp(−1v¯f(n)+Δv¯f)exp−αv∗v¯fexp−ΔGmkBT[1(v¯f(n)+Δv¯f)βS(cosh(σDP(n)+ΔσDPσ0)−1)−1nD+κ∇2v¯f(n)]=0

the initial values ($\Delta {{\bar{v}}_{f}}^{0},\Delta {\sigma_{DP}}^{0}$) are taken as the solution from the previous step. In the Newton–Raphson method, the solution ($\Delta {{\bar{v}}_{f}}^{k},\Delta {\sigma_{DP}}^{k}$) at the ***n***th step will be updated for the calculation ($\Delta {{\bar{v}}_{f}}^{k+1},\Delta {\sigma_{DP}}^{k+1}$) in the next step. Trial stress tensor is $\sigma_{DP,(n+1)}^{trial}$. The material stiffness matrix $D_{ijkl}^{ep}$ can also be updated. By solving the system of linear equations under a specific external load, we can obtain FV, strain and stress field. Analyzing the evolution of these key fields, we can obtain the microstructure evolution and the mechanical properties of BDMGCs with various particles. Each plane strain model consists of 30,000 square meshes and applies periodic boundary conditions. The tensile strain rate is set at 0.1/s.

## 3. Results and Discussion

**Figure 2:**
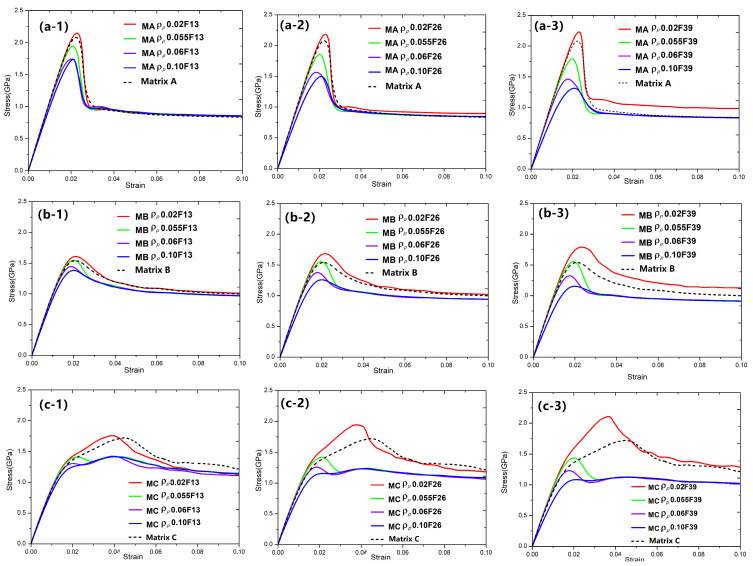
The stress–strain curves of BDMGCs containing the specific fraction *F* = 13% (-1), 26% (-2), and 39% (-3) of particles with $\rho_{p}$ = 0.02, 0.055, 0.06, and 0.10, and diameter *D* = 10 in matrices A (**a**), B (**b**), or C (**c**).

To obtain how the fraction and characteristics of particle reinforcements affect the mechanical properties of BDMGCs, in Figure 2 we first researched the effect of particles with $\rho_{p}=0.02,\;0.055,\;0.06,\;and\;0.10$ on the mechanical properties of BDMGCs which contain the specific fraction *F* = 13%, 26%, and 39% of particles with a diameter *D* = 10 in matrices A, B, or C. The stress–strain curves of the samples (“MA-*D*10*F*13”, “MA-*D*10*F*26”, “MA-*D*10*F*39”, “MB-*D*10*F*13”, “MB-*D*10*F*26”, “MB-*D*10*F*39”, “MC-*D*10*F*13”, “MC-*D*10*F*26”, and “MC-*D*10*F*39”) confirm that the particles with harder particles (with smaller values of FV) make the composite stronger (as indicated by the red solid lines in Figure 2) and softer particles (with larger value of FV) soften the composite (as indicated by the blue solid lines in Figure 2) as compared to that of MG matrix (as indicated by black dashed lines in Figure 2). This is indicated by changes in mechanical strength, including yield stress and peak stress. The yield stress is defined by the 0.2% strain offset method, while peak stress is the first occurrence of maximum stress. The flow stress is the asymptotic stress value at the large deformation strain. In Figure 2, the lower FV value $\rho_{p}$ of particles, the higher steady stress. This trend is enhanced with an increased volume fraction.

**Figure 3:**
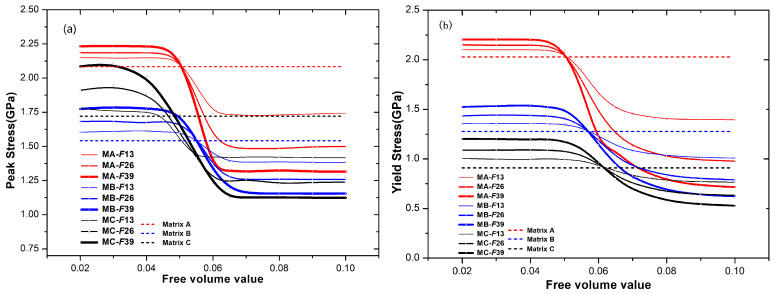
The peak stress (**a**) and yield stress (**b**) versus the varying FV value $\rho_{p}$ of particles with the given diameter (D = 10) and fractions in the certain matrix.

To explore the impact of fraction and characteristics of droplet reinforcement on the mechanical properties, we summarize the changes in the strength (both peak and yield stress) BDMGCs containing the varying FV value $\rho_{p}$ of particles with diameter *D* = 10 and fraction *F* = 13%, 26%, and 39% in matrices A, B, or C in Figure 3. As illustrated in Figure 3, the lower FV values ($\rho_{p}$) of particles enhance strengthening effects, when the $\rho_{p}$ is below certain threshold values closely related to the matrix type, the strengthening effect remains stable. Conversely, higher FV values lead to softening effects; when the $\rho_{p}$ exceeds these threshold values, the softening effect stabilizes. In Figure 3, one can observe that the threshold values are independent of volume fraction and depend on the matrix type. In Figure 3a, as the $\rho_{p}$ is less than 0.04 or greater than 0.065, the peak stress of BDMGCs with matrix A remains nearly constant; when the $\rho_{p}$ is less than 0.045 or greater than 0.07, the peak stress of BDMGCs with matrix B keep almost constant; when the $\rho_{p}$ is less than 0.03 or larger than 0.065, the peak stress of BDMGCs with matrix C continues to be nearly constant. In Figure 3b, when the particle density $\rho_{p}$ is less than 0.04 or greater than 0.09, the yield stress of BDMGCs with matrix A, B, or C keeps almost constant. One notes that the volume fraction typically enhances the effect of particle variety ($\rho_{p}$) usually. In Figure 3a, three curves of peak stress versus particle density FV values ($\rho_{p}$) in the specific matrix (for examples: “MA-*D10F13*”, “MA-*D*10*F*26”, or “MA-*D10F39*”) intersect at a single point. This indicates that a specific or critical FV value $\rho_{pc}$ will have minimal impact on the strength of BDMGCs as the fraction increases. The intersection FV values $\rho_{pc}$ of particles for the peak stress are 0.050, 0.055, and 0.055 in the matrix A, B, and C, while for yield stress they are 0.050, 0.057, and 0.061 in the matrices A, B, and C, respectively. The yield stress and peak stress of BDMGCs with particles $\rho_{pc}$ are identical to those of the monolithic matrices, except the peak stress of the system MC.

In Figure 2c, the BDMGCs with hard particles not only possess the increased strength but also the enhanced second hardening moduli. Comparing the samples “MC-*D10*$\rho_{p}$*0.02F13*”*,* “MC-*D*10$\rho_{p}$*0.02F*26”, and “MC-*D*10$\rho_{p}$*0.02F*39”, the strain hardening moduli (21.18 GPa, 28.3 GPa, and 35.77 GPa), peak stress (1.777 GPa, 1.921 GPa, and 2.085 GPa) and yield stress (1.004 GPa, 1.089 GPa, and 1.203 GPa) are almost increasing with the increase in volume fraction and are larger than those of matrix C: strain hardening modulus (20.28 GPa), peak stress (1.722 GPa) and yield stress (0.9089 GPa). Conversely, for the samples “MC-*D10*$\rho_{p}$*0.10F13*”, “MC-*D*10$\rho_{p}$*0.10F*26”, and “MC-*D*10$\rho_{p}$*0.10F*39”, their strain hardening moduli (9.817 GPa, 5.775 GPa, and 3.861 GPa), peak stresses (1.417 GPa,1.2376 GPa, and 1.124 GPa) and yield stresses (0.7655 GPa, 0.632 GPa, and 0.5283 GPa) are almost decreasing with the increase in volume fraction; these values are lower than those of matrix C. The stress–strain curve of sample “MC-*D*10$\rho_{p}$*0.10F*39” is almost the ideal elastoplastic.

**Figure 4:**
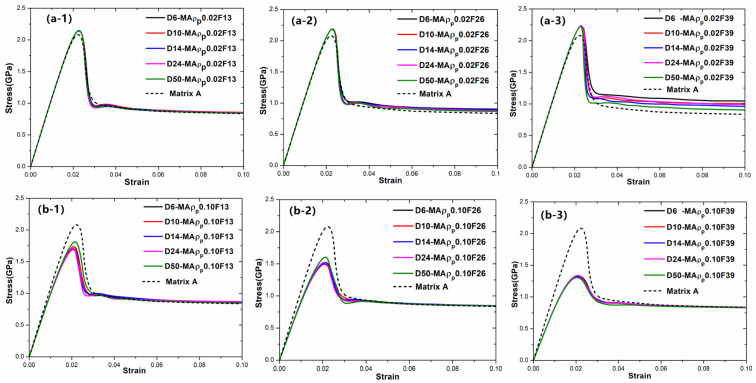
The tensile stress–strain curves of BDMGCs containing the specific fraction *F* = 13% (-1), 26% (-2), and 39% (-3) of particles with FV density $\rho_{p}$ = 0.02 (**a**), and 0.10 (**b**), along with diameter *D* = 6, 10, 14, 24, and 50 in matrix A.

**Figure 5:**
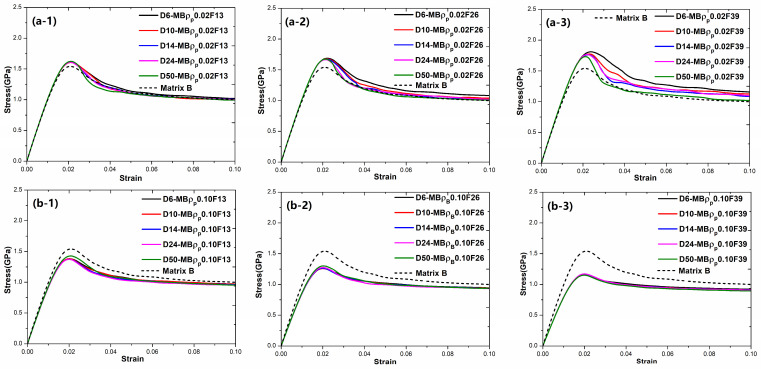
The tensile stress–strain curves of BDMGCs containing the specific fraction *F* = 13% (-1), 26% (-2), and 39% (-3) of particles with FV density $\rho_{p}$ = 0.02 (**a**), and 0.10 (**b**), along with diameter *D* = 6, 10, 14, 24, and 50 in matrix B.

**Figure 6:**
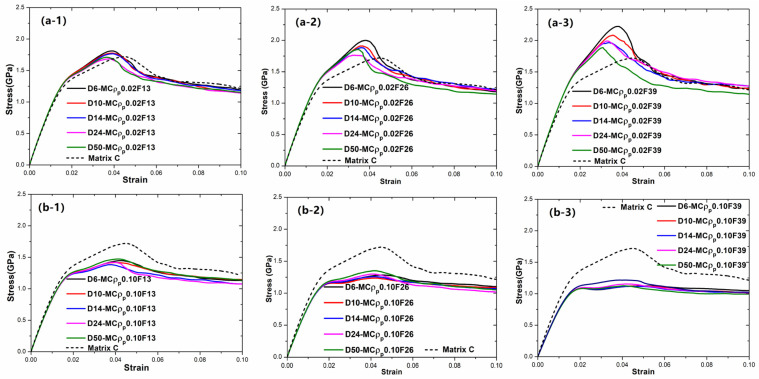
The tensile stress–strain curves of BDMGCs containing the specific fraction *F* = 13% (-1), 26% (-2), and 39% (-3) of particles with FV density $\rho_{p}$ = 0.02 (**a**), and 0.10 (**b**), along with diameter *D* = 6, 10, 14, 24, and 50 in matrix C.

To investigate the influence of particle size and matrix on the mechanical properties of BDMGCs, the tensile stress–strain curves of systems BDMGCs containing the varying fraction (*F* = 13%, 26%, and 39%) of hard particles ($\rho_{p}$ = 0.02) and soft particles ($\rho_{p}$ = 0.10), along with diameters D = 6, 10, 14, 24, and 50 in matrices A, B, and C are shown in Figure 4, Figure 5, and Figure 6, respectively. In Figure 4(a-1), one can see the peak stress remains constant as the hard ($\rho_{p}$ = 0.02) particle diameter increases, with a fixed fraction of 13%. In Figure 4(a-1–a-3), the peak stress increases with the increasing fraction and exceeds that of matrix A. In Figure 4(a-3), the flow stress decreases significantly with decreasing diameter at a fraction of 39%. However, at a lower fraction of 13%, the flow stress changes very little with varying diameter. In Figure 4(b-1), one can see the peak stress increases as the soft ($\rho_{p}$ = 0.10) particle diameter increases, with a fixed fraction of 13%. In Figure 4(b-1–b-3), the change in peak stress decreases with increasing fraction, while flow stress remains nearly constant and is equivalent to that of matrix A. In Figure 5(a-1–a-3), the peak stress increases with the increasing fraction and exceeds that of matrix B. In Figure 5(a-3), both peak stress and flow stress increases significantly as the hard ($\rho_{p}$ = 0.02) particle diameter decreases, remaining higher than those of matrix B. In Figure 5(b-1–b-3), the change in flow stress keeps constant as the soft particle ($\rho_{p}$ = 0.10) diameter increases at a fixed fraction. As the fraction increases, flow stress decreases and falls below that of matrix B. In Figure 6(a-1–a-3), BDMGCs with the hard particle ($\rho_{p}$ = 0.02) show increased strain hardening moduli and peak stresses at the higher fractions and smaller diameter, exceeding those of matrix C. In contrast, in Figure 6(b-1–b-3), BDMGCs with the soft particle ($\rho_{p}$ = 0.10) exhibit decreased strain hardening moduli and peak stresses at higher fractions.

**Figure 7:**
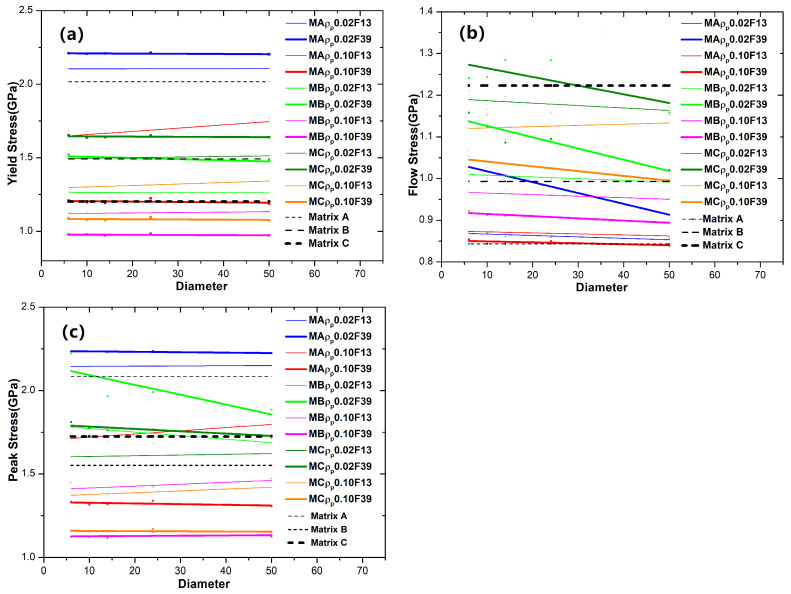
The mechanical strengths (yield stress (**a**), flow stress (**b**) and peak stress (**c**)) of BDMGCs versus the diameter or size of particles. The red, magenta, and orange lines are for GNMGCs samples with soft particles ($\rho_{p}$ = 0.10) and the blue, green, and olive lines are for GNMGCs samples with hard particles ($\rho_{p}$ = 0.02). The black dashed lines with the legend of “Matrix” are for the corresponding monolithic MGs.

The general trend of the change in strength (yield stress, flow stress, and peak stress) versus the particle size can be extracted in Figure 7. Compared to the corresponding MG matrix, hard particles increase the strength (yield and peak stresses), while soft particles decrease it. This effect becomes more pronounced with a higher particle fraction. As shown in Figure 7a, one can see the yield stress of all GNMGCs samples with hard particles quantitatively remains constant as the particle size increases, while samples with soft particles show an increase in yield stress at larger particle sizes, particularly at low fractions. In Figure 7b, the flow stress of GNMGCs samples increases as particle size or diameter decreases, with this trend becoming more pronounced at higher particle fractions. In Figure 7c, the peak stress of all GNMGCs samples with hard particles increases as particle size or diameter decreases, while for samples with soft particles, it increases as particle size or diameter increases.

**Figure 8:**
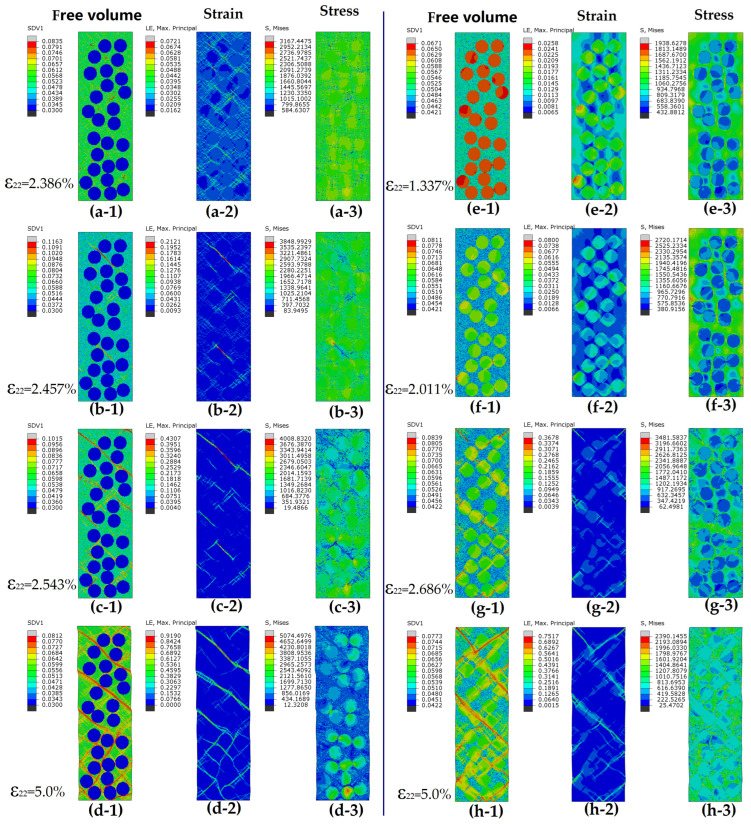
Contour plots for free volume (-1), shear strain (-2), and shear stress (-3) of sample “MA-*D24*$\rho_{p}$*0.03F*39” with hard particles ($\rho_{B}$ = 0.03) at strains 0.02386 (**a**), 0.02457 (**b**), 0.02543 (**c**), and 0.05 (**d**), and sample “MA-*D*24$\rho_{p}$*0.10F*39” with soft particles ($\rho_{p}$ = 0.10) at strains 0.01337 (**e**), 0.02011 (**f**), 0.02686 (**g**), and 0.05 (**h**). The color bar is shown on the left of each figure.

The fraction, size, and characteristics of particle reinforcements could affect BDMGCs performance by influencing the nucleation, formation, and propagation of SBs in the MG matrix. The mechanisms for tuning mechanical performances should involve stress fields in the matrix. To explore the new deformation mechanisms of BDMGCs, we analyze the spatial distributions of FV, strain field, and stress field under various strain states to understand local shear behaviors. To obtain the details of shear banding, the contour maps of FV, strain, and stress of sample “MA-*D24*$\rho_{p}$*0.03F*39” with hard particles ($\rho_{B}$ = 0.03) at strains 0.02386, 0.02457, 0.02543, and 0.0500, and sample “MA-*D*24$\rho_{p}$*0.10F*39” with soft particles ($\rho_{B}$ = 0.10) at strains 0.01337, 0.02011, 0.02686, and 0.0500 are shown in Figure 8. For the sample *D*24$\rho_{p}0.03$*F*39 with hard particles ($\rho_{p}$ = 0.03), the yield stress of particles is much higher than that of the matrix. Before yielding of matrix, particles maintain higher stress and less strain than the matrix for the mismatch of mechanical property. In Figure 8a, the strain and stress around the particle are higher than those in other areas of the matrix, especially for the shear banding zone along 45° with respect to the uniaxial loading direction. In the shear banding zone around a particle it is variable and fluctuating. Additionally, the interaction of multiple droplets complicates the stress field in the surrounding extended zone. As shown in Figure 8b, when the applied stress at weak points exceeds their yield strength, the main SBs will initiate and nucleate around particles in that zone. As the applied stress reaches high enough to break down the matrix, the SBs will develop and propagate on the direction of maximum effective stress (Figure 8c). During the propagation of SBs, the head region of SBs will be the stress concentration point; after the propagation of SBs, the local strain and FV on the previous head of SBs will increase quickly and then decrease slowly; however, the stress will abruptly drop. The deformation regions of SBs are still restricted to their original location although more new deformed regions are created elsewhere including those with larger values of FVs along the propagation path. During the shear banding, some SBs will meet and be stopped by the harder particles and the probability of encounter closely relates to the fraction and distribution of isolated particles. Based on the FV gradient effect, the stopped SBs are required to store more energy and larger stress to break the gradient interfaces between the particles and matrix. The method requiring less energy consumption or the easier way to propagation is meandering and deflecting along the tangential direction of the particles. The deflection of main SBs prevents the breakdown of hard particles, while the particles with extreme low FV barely interact with the main SBs during propagations in the matrix. The detour and deflection of SBs extend the propagating distance of SBs (Figure 8d) and then lead to the strengthening and toughening of BDMGCs. The localized deformation zones spread more widely. The stop and detour processes in the matrix occur and correspond to the peak stress dropping into a steady state on the stress–strain curves. One can see that the smaller FV of the particles is, the higher the steady stress is; the higher the fraction of hard particles with a given diameter is, the higher the steady stress is. As shown in Figure 3, when the FV of particles falls below certain threshold values associated with the matrix type, the strengthening effect remains stable, and both yield strength and peak stress exhibit minimal variation. These phenomena arise from the initiation and propagation of all SBs in the matrix. The initiation points of SBs are typically distanced from hard particles, which have minimal influence on far-field stress as the FV in hard particles decrease to threshold values. As SBs encounter hard particles, they will detour and deflect; however, the concentrated stress at the leading edges of these SBs is insufficient to alter the microstructure of hard particles when their FV values of B are less than the threshold values related to the matrix type. As the fraction and FV of particles and the matrix species are provided, it can be suggested that smaller particle radius leads to higher steady and peak stress.

For the sample *D*24$\rho_{p}0.10$*F*39 with soft particles ($\rho_{p}$ = 0.10), the yield stress of particles is significantly lower than that of the matrix. Before the initiation and nucleation of main SBs, the FV of particles quickly annihilate and evolve. The minor SBs will quickly initiate and nucleate in the glassy particles; however, they are trapped and restricted on the particles. As shown in Figure 2b, the decrease in modulus and peak stress is results from the strain softening of those minor SBs. Under the loading conditions, the strain of particles is higher than that of the matrix, but the effective stress of particles is lower (Figure 8e). The stress field in the matrix surrounding one particle is heterogeneous. In Figure 8f, there are two groups of stress concentration regions in the matrix: the one (about 90° or 0° off the loading axis) makes contact with the particles; the others do not make contact with the particles and are located in the middle region between two particles whose center line is about 45° off the loading axis. The coupling stress fields in the matrix are caused due to randomly distributed soft particles that are more complicated and heterogeneous. The main SBs typically nucleate and initiate at the second stress concentration region (Figure 8g); for some special distribution of particles, few of the main SBs can start at the first region. With continued loading, the nucleation of SBs grows into the mature ones that propagate along the direction of maximum equivalent shear stress. The mature SBs pass through the soft particles (Figure 8h). One can see that the width of propagation of main SBs are varies as they cross the interface between the matrix and particles, the local stress and strain of SBs in the particle region will release suddenly. The soft particle region acts as an energy or stress buffer for SBs traveling through the particles. Several main SBs connect and traverse through the cross-section. In Figure 3, when the FV of particles exceeds certain threshold values associated with the matrix type, the softening effect remains stable, and the strength (yield and peak stress) remains nearly constant. Before mature SBs initiate, larger soft particles quickly annihilate, reducing their FV to a specific level that affects yield stress. Most of the mature SBs initiate at the stress concentration region where they are less influenced by soft particles and intersect with those whose microstructure has evolved with specific FVs. Particularly, it was deduced that the inclusions undergo a phase transformation (the soft phase transforms into the harder phase), the propagation of main SBs in the matrix is buffered and then subsequently hampered. This leads to the enhancement of the mechanical property of BDMGCs.

The matrix is fundamental to the mechanical properties of BDMGCs and the particles with various mechanical property (soft or hard) should have different mechanisms to alter the shear banding and to tune and enhance the plasticity and strength of BDMGCs. A specific or critical FV value $\rho_{pc}$ will have minimal impact on the strength of BDMGCs as the fraction increases. Mature SBs typically initiate at the interfaces between particles and matrix. The stress in a particle equals the mean stress in the matrix, meaning that particles with the critical FV value minimally affect spatial stress in the matrix. SBs can easily traverse the particle zone, thus not altering the strength of BDMGCs. In the systems MC with particles, the process of main SBs is similar with the systems MA. The statistical variance of matrix C is greater than that of matrix A. Consequently, the deformations SBs in matrix C appear rugged and zigzagged, unlike the smooth and straight ones in matrix A. The region of stress concentration in matrix C comprises discrete concentration points which are located on the soft points of the heterogeneous matrix. The interaction (stop and detour) of SBs with hard particles can increase the strain hardening modulus, peak stress, and steady stress.

During the propagation of SBs, some but not all SBs will meet and be stopped by the hard particles and the probability of encounter closely related with the fraction and distribution of isolated particles. As the number of hard particles increases, SBs are more likely to detour and stop, leading to stronger interactions with the hard particles. The quantity of hard particles is closely related to particle diameter and volume fraction. The particle spacing in the matrix should affect the process of SBs. The Cu-Zr-Al metallic glass composites with the nanocrystals exhibit 0.5% tensile ductility, attributed to the precipitation of nanocrystals that alter the stress field and hinder and detour the propagation of SBs. Once the volume fraction of crystals and average particle size are known, the mean interparticle spacings or distances Γ can be calculated in three-dimension by [47]:

(15) $$\Gamma =\sqrt[{3}]{\frac{\pi }{6F}}D$$

**Figure 9:**
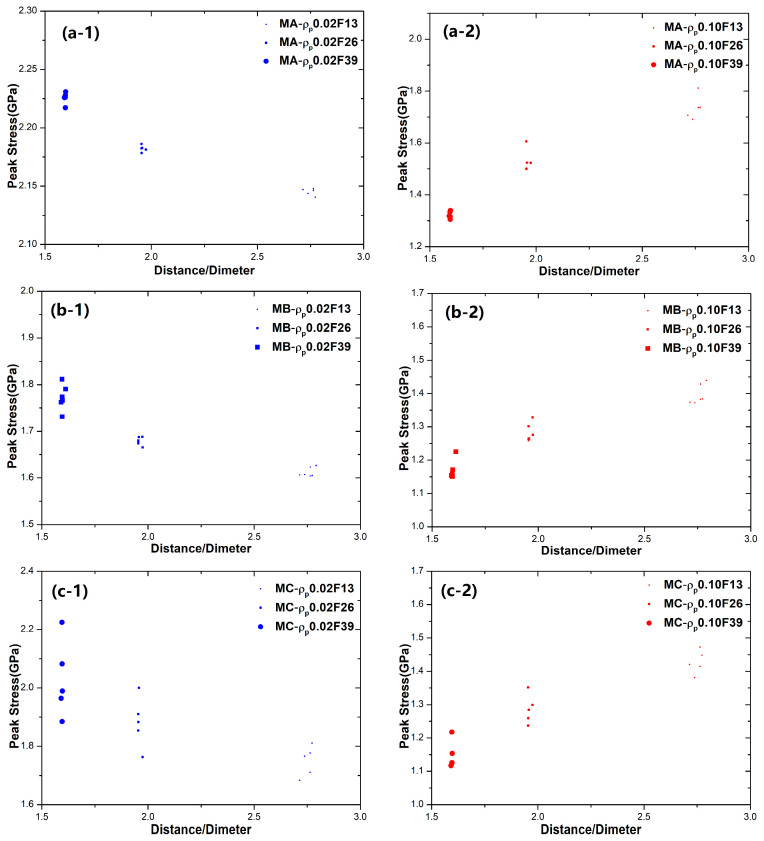
The peak stress versus the parameter *K* (particle distances/diameter). The blue dots indicate GNMGCs with hard particles ($\rho_{p}$ = 0.02) (-1), while the red dots represent GNMGCs samples with soft particles ($\rho_{p}$ = 0.10) (-2) in matrix A (**a**), B (**b**), and C (**c**).

To investigate the synergistic effect of particles (with various fraction, size, and type of particle reinforcements) and the matrix, in Figure 9, we summarize and extract the peak stress of all BDMGCs in relation to the parameter *K* (interparticle distances divide diameter). For the BDMGCs with hard particles, it was observed that the peak stress increases as the parameter *K* decreases; for the BDMGCs with soft particles, peak stress increases as the parameter *K* increases. In the BDMGCs, the size effect of particles within a heterogeneous matrix is more significant than that of particles in a homogeneous matrix.

Here in models of BDMGCs, the isolated hard particles have a very low efficiency when intercepting SB, due to the detour deflection of SBs. It could be suggested that the new merging and percolated patterns of particles or inclusion are more effective at stopping and hindering the propagation of the main SBs than the isolated hard particles. The morphology could serve as a key parameter to affect the properties of MGCs. BDMGCs with isolated soft particles can redirect the direction of SBs and reduce the strength of BDMGCs. It follows that the soft particle undergoing phase transformation (the soft phase transforms into the harder phase) can effectively capture and stop the mature SBs, thereby strengthening and toughening the mechanical property of BDMGCs.

## 4. Conclusions

We investigate the mechanical properties of BDMGCs with various characteristics of particle reinforcements, quantitatively examining their influences on the mechanical properties and the deformation mechanisms. The mechanism of hard and soft particles in tuning and adjusting the properties is attributed to effective stress fields generated by the particles within the matrix, which affect both the initiation and propagation of SBs. In the BDMGCs containing the hard particle, the SBs usually initiate within the matrix before propagating into mature SBs along the direction of maximum effective stress. The initiation location for SBs formation is random due to the stochastic distribution of hard particles. The stop and obstacle of SBs occur when they encounter hard particles, leading to an increase in strength. During the propagation of SBs, some but not all SBs meet and are subsequently stopped by the hard particles. An increased fraction of hard particles increases the likelihood that SBs will encounter obstacles. Conversely, for the BDMGCs samples containing soft particles, the deformation initially occurs in the soft particle zone. The minor SBs rapidly initiate and nucleate in the glassy particles; however, they are trapped and constrained within these softer inclusions. The main SBs generally occur in regions situated between two soft particles whose center line is about 45° off the loading axis. These main SBs propagate through and traverse across the soft particle domains, connecting with each other and traversing the cross-section of samples.

The synergistic effect of reinforcements and inhomogeneous matrix requires more attentions. We demonstrate that the heterogeneous microstructure of BDMGCs influences SB propagation. Based on the micromechanics of BDMGCs, we provide foundational insights for designing BDMGCs with heterogeneous microstructures. Here in models of BDMGCs, it is found that increasing the fraction of reinforcement is a simple and effective way to enhance their mechanical properties. The isolated hard particles are inefficient at intercepting SBs; while smaller hard reinforcements with a long strip shape will improve the strength and toughness of BDMGCs. The soft particle undergoing phase transformation (from the soft phase to the harder phase) can effectively capture and halt the mature SBs, thereby strengthening and toughening BDMGCs. Additionally, in our future work, we will explore and investigate the effects arising from heterogeneous reinforcements which have percolated patterns or exhibit phase transformation. The quantitative studies demonstrates that BDMGCs can be optimized by tuning the heterogeneous microstructures and properties of reinforcements.

## Data Availability

The raw data supporting the conclusions of this article will be made available by the authors on request.
